# Textbooks authors, publishers, formats and costs in higher education

**DOI:** 10.1186/s13104-019-4099-1

**Published:** 2019-01-24

**Authors:** Eamon Costello, Richard Bolger

**Affiliations:** Dublin City University, Glasnevin, Dublin 9, Ireland

**Keywords:** Textbooks, Higher education, Open textbooks, Open education, Open datasets, Open science

## Abstract

**Objectives:**

There is little empirical data reported on retail prices of college textbooks beyond self-reported surveys and no published datasets. Textbooks, as an ancillary cost, can contribute to the overall rising cost of education which can impact upon students’ ability to succeed in Higher Education. This study sought to understand more about costs of college textbooks by conducting a systematic collection of several thousand textbooks from faculty readings lists in one Higher Education Institution in Ireland and a retrieval and analysis of the retail prices of a selection of those books.

**Data description:**

Queries were made of the course catalogue database of a Higher Education Institution in Ireland resulting in generation of records for required and recommended textbooks for 15,414 books from 3030 unique courses for the academic year 2017–2018. This data was cleaned and processed before being used to query Google Books API. The dataset presented here represents the combination of data from the course catalogue and the Google Books API queries and comprises 2940 records of textbooks. Details for each book including title, authors, publisher, ISBN, retail price, ebook format, pdf availability, and public domain availability.

## Objective

There is little empirical data reported on retail prices of college textbooks beyond self-reported surveys [1–3] and no published datasets. Textbooks, as an ancillary cost, can contribute to the overall cost of education which is rising and can seriously impact upon students’ ability to succeed in Higher Education [4]. This study sought to understand more about costs of college textbooks by conducting a systematic collection of several thousand textbooks from faculty readings lists in one Higher Education Institution in Ireland and a retrieval and analysis of the retail prices of a selection of those books [5, 6].

An analysis and discussion of the implications of the dataset are published elsewhere including extrapolations to show the likely full economic costings of books on average per student for their studies [8]. Other research has been conducted on college textbooks such as examining whether there is a gender bias in booklists [9]. Our dataset is considerably larger than those considered by such studies to date and hence we hope could be of use to other researchers.

## Data description

This dataset comprises meta-data on textbooks from readings lists from one Higher Education Institution in Ireland. The data comprises 2940 records each representing a textbook (from ~ 578 courses). The institution has a student population of over 10,000. Each record in the dataset corresponds to a book. Each book has: one or more authors, a publisher, an 11 digit ISBN, a 13 digit ISBN, a url pointing to a thumbnail image of the book, an indicator of whether the book is available in an ePub version, an indicator as to whether the book is the public domain, and an indicator as to whether the book is available in a PDF version. The complete records are available as a JSON file made available with this article. See SampleRecord1.js for an example of one JSON record.

As per Data file 6, 1168 (40%) books have either a PDF or ebook version. 1219 (39.7%) have a PDF version and 1442 (34.65%) have an ebook version. 6 (0.18%) books have a public domain license. As per Data file 7, 596 (20%) of books have a retail price in US dollars. The prices range from $0.99 to $452. The mode of the retail price of a book is $9.99, the mean price is $56.67 and the median is $40. 2867 books have one or more discernable authors. The distribution of the number of authors per book is given in Data file 8.

The data was derived from two sources. The first source was an electronic course catalogue containing the recommended and required readings for each course in one Higher Education Institution in Ireland with a student population of over 10,000. The catalogue was queried using SQL queries (Data file 3). This first set of data (Data file 1) comprised textbook details for 15,414 books from 3030 unique courses for the academic year 2017–2018. This data was then combined with data from Google Books which contains data on over 30 million books from its own bookstore and a network of resellers (Data file 2). The Google Books API [7] was queried using the Google Cloud Computing platform, specifically a custom written JavaScript program deployed as middleware via Google Cloud Functions: see the figure in Data file 9 for a schematic overview. Google Books API returned details on retail prices, book formats and public domain availability. In addition, it improved the data on publisher, ISBN and author as the data from the course catalogue was originally manually entered by lecturers and contained errors. Finally, we loaded the returned JSON into a document store (MongoDB) for querying and analysis.

Table 1 provides detailed links to all the data described in this article.

**Table 1:** Overview of data files/data sets

Label	Name of data file/data set	File types (file extension)	Data repository and identifier (DOI or accession number)
Data file 1	allBooks.json	JSON (json)	Zenodo 10.5281/zenodo.1489526
Data file 2	books.json	JSON (json)	10.5281/zenodo.1489526
Data file 3	selectModuleBooks.sql	SQL (sql)	10.5281/zenodo.1489526
Data file 4	getData.js	JavaScript (js)	10.5281/zenodo.1489526
Data file 5	Figure1	PNG (png)	10.5281/zenodo.1489526
Data file 6	Figure2	PNG (png)	10.5281/zenodo.1489526
Data file 7	Figure3	PNG (png)	10.5281/zenodo.1489526
Data file 8	Figure4	PNG (png)	10.5281/zenodo.1489526
Data file 9	Figure5	PNG (png)	10.5281/zenodo.1489526

## Limitations

Google Books has known limitations and does not provide comprehensive coverage of all books [10]. Its indexation policies and coverage rules are not released by Google.

## Abbreviations

SQL: Structured Query Language
JSON: JavaScript Object Notation
